# Neurological, psychological, psychosocial complications of long-COVID and their management

**DOI:** 10.1007/s10072-024-07854-5

**Published:** 2024-11-09

**Authors:** Sareesh Naduvil Narayanan, Sreeshma Padiyath, Krishnapriya Chandrababu, Lima Raj, Baby Chakrapani P. S., George Abraham Ninan, Ajith Sivadasan, Alexander Ryan Jacobs, Yan Wa Li, Anand Bhaskar

**Affiliations:** 1 Department of Physiology, School of Medicine and Dentistry, AUC-UK Track, University of Central Lancashire, Preston, UK; 2 Department of Microbiology, School of Medicine and Dentistry, AUC-UK Track, University of Central Lancashire, Preston, UK; 3 Centre for Neuroscience, Department of Biotechnology, Cochin University of Science and Technology (CUSAT), Kochi, India; 4 Department of Psychology, Sree Sankaracharya University of Sanskrit, Kalady, India; 5 Centre for Excellence in Neurodegeneration and Brain Health (CENABH), Cochin University of Science and Technology (CUSAT), Kochi, India; 6 Department of Neurology, Christian Medical College (CMC), Vellore, India; 7 School of Medicine and Dentistry, AUC-UK Track, University of Central Lancashire, Preston, UK; 8 Faculty of Medicine, Macau University of Science and Technology, Macau, China; 9 Department of Physiology, Christian Medical College (CMC), Vellore, India

**Keywords:** COVID-19, Cytokine storm, Long-COVID, Sleep disturbance, Memory impairment, Anxiety, Neurodegeneration

## Abstract

Since it first appeared, Severe Acute Respiratory Syndrome Coronavirus 2 (SARS-CoV-2) has had a significant and lasting negative impact on the health and economies of millions of individuals all over the globe. At the level of individual health too, many patients are not recovering fully and experiencing a long-term condition now commonly termed ‘long-COVID’. Long-COVID is a collection of symptoms which must last more than 12 weeks following initial COVID infection, and which cannot be adequately explained by alternate diagnoses. The neurological and psychosocial impact of long-COVID is itself now a global health crisis and therefore preventing, diagnosing, and managing these patients is of paramount importance. This review focuses primarily on: neurological functioning deficits; mental health impacts; long-term mood problems; and associated psychosocial issues, among patients suffering from long-COVID with an eye towards the neurological basis of these symptoms. A concise account of the clinical relevance of the neurological and psychosocial impacts of long-COVID, the effects on long-term morbidity, and varied approaches in managing patients with significant chronic neurological symptoms and conditions was extracted from the literature, analysed and reported. A comprehensive account of plausible pathophysiological mechanisms involved in the development of long-COVID, its management, and future research needs have been discussed.

## Introduction

COVID-19 has had a significant negative impact on the health and economy of millions of individuals around the world. Globally, as of April 2024, approximately 775 million confirmed COVID-19 cases have been reported, including > 7 million deaths [1]. It is also worth noting that as of April 2024, a total of 13.9 billion vaccine doses have been administered globally. A situation analysis by WHO by region demonstrates that COVID-19 confirmed cases are significantly high in Europe, the Western Pacific, the Americas, and the South-East when compared to Asia, the Eastern Mediterranean, and Africa [1].

Although the initial focus during this pandemic was to overcome the death toll, it has now become apparent that many of the patients have been left unable to overcome the devastating long-term effects of this disease and that they are not fully recovering. A new term has emerged to describe this phenomenon, ‘long-COVID’ [2, 3]. Long-COVID was initially proposed to be classifiable as one of two types. First, the ongoing symptomatic COVID wherein the COVID-19 symptoms continue to be present for an extended period of 4 to 12 weeks. Second, post-COVID syndrome wherein some COVID-19 symptoms continue to persist after 12 weeks, sometimes well beyond. However, long-COVID is now emerging to be understood as a collection of multiple symptoms lasting > 3 months after the first COVID infection that cannot be adequately explained by alternative diagnosis. There is a wide but defined constellation of symptoms associated with this condition [4, 5]. Typically, long-COVID symptoms vary substantially by person, starting from breathing difficulties, chest problems, but also include other common problems such as fatigue, fever, pain, neurological deficits including difficulty focusing, headaches, problems sleeping, tingling & numbness, dizziness, delirium, systemic symptoms such as ear, nose, and throat problems, dermatological problems, stomach problems, musculoskeletal problems, and sustained mental health and mood dysregulations including anxiety, worry and depression. It is now clear that in at least some percentage of the infected population, long-COVID is a chronic condition [6]. Taking into consideration the high number of COVID-19 cases in certain geographical locations that struggle to get access to healthcare, a good number of patients undoubtedly will be affected by this devastating condition without ever being diagnosed. As many of the most devastating of these described symptoms are neurological and psychological in nature, it is of paramount importance to study and understand the neurological and psychological consequences of long-COVID.

A concise account of the clinical relevance of the neurological and psychosocial impacts of long-COVID, it’s long-term morbidity, and varied approaches in managing patients with significant chronic neurological symptoms and conditions were extracted from the literature, analysed, and reported. Specifically, this review focuses on long-COVID associated *a)* neurological complications such as brain fog, sleep disturbances, memory problems, and neurodegeneration, *b)* problems in patients suffering from Alzheimer’s and Parkinson’s diseases, *c)* psychological complications such as anxiety, worry, depression and quality of life, *d)* pathophysiological mechanisms leading to neurological and psychological symptoms, and their *e)* clinical management.

## Methods

### Search strategy and databases used

Commonly accessed and broadly utilised electronic databases such as MEDLINE/PubMed, and SCOPUS, were the primary databases used for searching published literature. The keywords used were as follows. “COVID-19/Novel coronavirus and Neuroinvasion”, “Long-COVID and Brain fog/Sleep disturbances/Memory impairment/Neurodegeneration/Alzheimer’s disease/Parkinson’s disease, Long-COVID and Psychological changes/Psychosocial effects/Quality of life/Anxiety/Depression/Altered mental status/Gut dysbiosis”, “Long-COVID and Pathophysiology/Management”. Article titles and abstracts were initially screened, and duplicate records were removed at this stage itself. The remaining articles were assessed for their eligibility to be included in this review.

### Inclusion criteria

The inclusion criteria were peer-reviewed original research on long-COVID including clinical, basic research, and related systematic reviews. Resources available in the public domain from major regulatory bodies such as WHO and CDC were also referred to if they contained information relevant to various sections of this review.

### Exclusion criteria

Studies excluded from the analysis are articles not officially published in a peer-reviewed journal, conference abstracts and proceedings, corrigendum documents, repeated publications, unrelated articles like acute neurological effects of COVID-19, and non-English articles.

### Qualitative synthesis

Full text of each of the articles selected for qualitative synthesis (*n* = 228) was thoroughly analysed. Four major areas of outcome measure were identified, and they were neurological, psychological, and psychosocial complications of long-COVID and management of neurological complications in long-COVID. Two authors have worked independently to extract data/information from reports deemed to be included in each of the sections of this review. Any conflicts between these authors’ findings were resolved by discussion and consensus in the presence of the third author. Major neurological, psychological, and psychosocial complications seen in long-COVID patients were extracted and summarised alongside suggesting possible underlying pathophysiological mechanisms for these complications and their management.

### Neuroinvasion of SARS-CoV-2 and its implications in the development of long-COVID

#### ACE2 expression in the brain

**Fig. 1:**
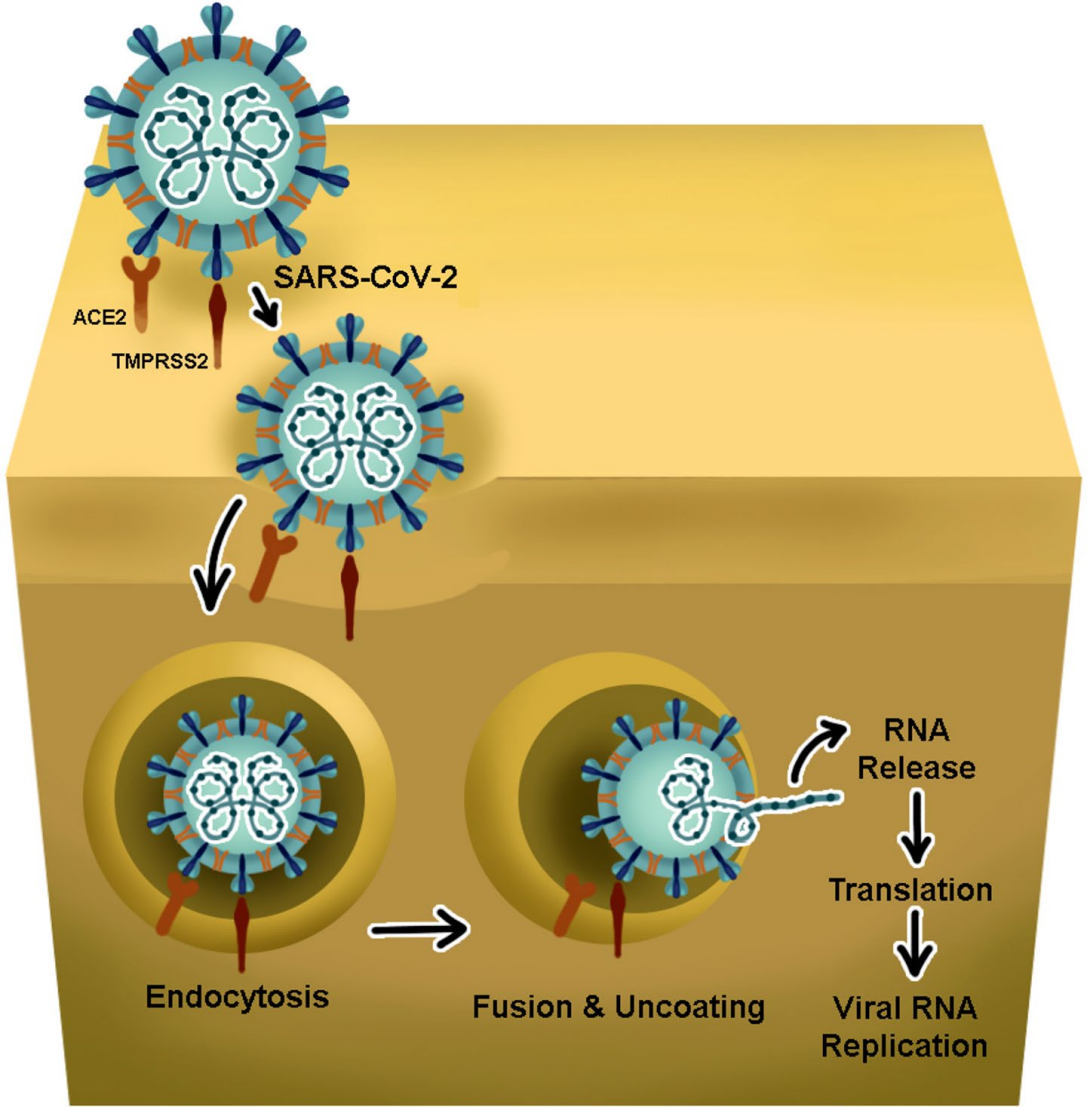
Schematic diagram showing the replication cycle of coronavirus. SARS-CoV-2 binds to the ACE2 receptor on the host cell via the receptor-binding domain of its spike protein. As depicted, TMPRSS2 is the host factor that promotes viral uptake and membrane fusion. The viral uncoating and release of RNA to the host cell cytoplasm results in immediate translation followed by viral RNA replication

ACE2, a transmembrane glycoprotein, is the key entry receptor for SARS-CoV-2 and plays a vital role in its pathogenesis. Extensive expression of ACE2 has been found on the surface of many tissues, including the respiratory system, cardiovascular system, gastrointestinal tract, kidneys, choroid plexus, testes, placenta, and bladder [7]. In the respiratory system, ACE2 is highly expressed in olfactory, nasal, bronchial epithelial cells, and type II alveolar epithelial cells [8]. In the cardiovascular system, ACE2 is expressed in myocytes, vascular endothelial cells, vascular smooth muscle cells of arteries, and venules [7]. In the brain, ACE2 is expressed in specific areas including the substantia nigra, brain ventricles, middle temporal gyri (excitatory and inhibitory neurons), and the posterior cingulate cortex. High expression of ACE2 has been reported in the posterior hypothalamic area, paraventricular nuclei of the thalamus, lateral hypothalamic area, paraventricular nuclei of the hypothalamus, piriform cortex, amygdala-hippocampal transition area, fastigial nucleus and hippocampal CA2 field [9, 10]. SARS-CoV-2 binds to the ACE2 receptor on the host cell via the receptor-binding domain of its spike protein. TMPRSS2 is the host factor that promotes viral uptake and membrane fusion. Viral uncoating and release of RNA to the cytoplasm leads to immediate translation. The resulting polyprotein is processed into both non-structural and structural proteins. Structural proteins translocate into the endoplasmic reticulum membrane. The new virion is then released from the infected cells by exocytosis (Fig. 1) [11].

#### Possible mechanisms of SARS-CoV-2 invasion to the nervous system

**Fig. 2:**
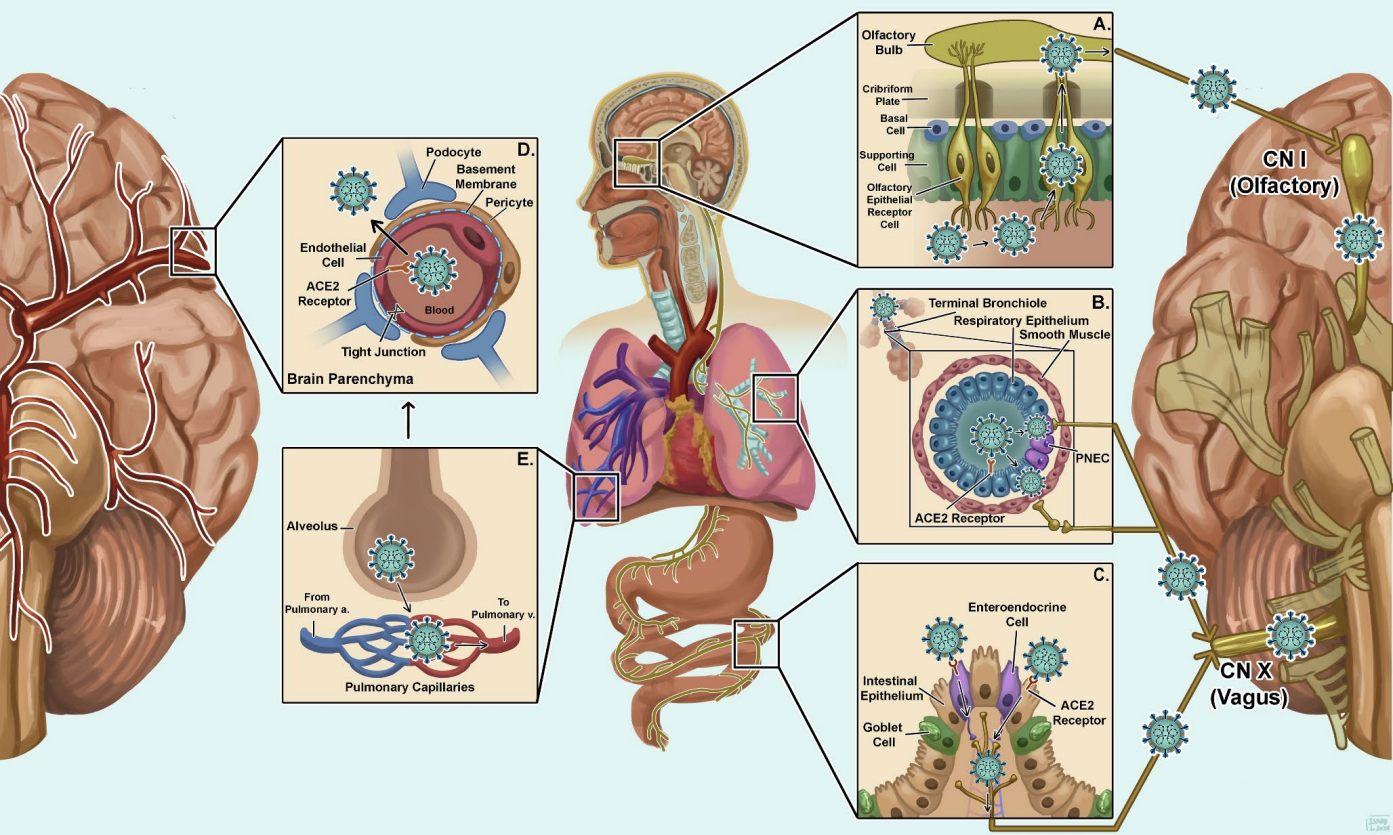
Pathways through which SARS-CoV-2 enters various cells including the nervous system. (**A**) Olfactory route via, olfactory receptor and CN-I, (**B**) Bronchial route via, CN-X, (**C**) Intestinal route via, CN-X, (**D**) Hematogenous route via, altered BBB to the brain parenchyma, (**E**) Hematogenous route via, infected alveolar parenchyma

Neuroinvasion of SARS-CoV-2 and triggered inflammation has been demonstrated by experimental animal models, organoid models, and autopsy studies [12, 13]. SARS-CoV-2 primarily replicates inside the respiratory tract (Fig. 1), however, this tract is innervated by several cranial nerves and these nerves are suggested to play a role in neuroinvasion (Fig. 2). Various studies have reported evidence of SARS-CoV-2 entry to CNS via the olfactory nerve, trigeminal nerve, or vagus nerve endings utilising retrograde transport mechanisms (Fig. 2). The virus also appears to neuroinvade by entering the blood from the lung, with the resulting viremia subsequently crossing the blood brain barrier (BBB) or the blood cerebrospinal fluid barrier; the choroid plexus [14–16] (Fig. 2).

#### Olfactory route

Retrograde dissemination of SARS-CoV-2 through the olfactory nerve seems to be one of the possible mechanisms of neuroinvasion. ACE2, TMPRSS2, and neuropilins are highly expressed in the olfactory epithelium, which makes it a key early infection site for CNS invasion [17, 18]. The virus may enter the brain through the olfactory mucosa neuroepithelium to the olfactory bulb, mitral cells, and olfactory nerve and from there it could spread to different areas of the brain [17]. An autopsy study demonstrated highest level of viral load in olfactory mucosa sampled directly beneath the cribriform plate. Additionally, viral RNA was detected in the olfactory bulb, trigeminal ganglion, and medulla oblongata [14]. Another study provided evidence for the involvement of neuropilin 1(NRP1) in SARS-CoV-2 entry into olfactory epithelium, neurons, and blood vessels of the cortex [18]. A non-human primate model study in rhesus monkeys found evidence of viral RNA in nasal mucosa, CSF, the olfactory trigone, and the entorhinal area by qRT-PCR following intranasal inoculation. These facts strongly support SARS-CoV-2 neuroinvasion via the olfactory route [15].

#### Vagus nerve route

Another possible SARS-CoV-2 spread into the CNS occurs through the vagus nerve of the lungs and gut afferents. SARS-CoV-2 neuroinvasion has been reported from the lungs via the vagus nerve to the autonomic nerve centres in the brain stem [19]. A human study revealed that cough, dizziness, inappropriate sinus tachycardia, and gastrointestinal symptoms were associated with vagus nerve damage [20]. Similarly, the role of the gastrointestinal tract in SARS-CoV-2 neuroinvasion has been proposed. The virus also invades through the gastrointestinal system and reaches the CNS directly through afferent fibres of the intestinal vagus nerve [21].

#### Hematogenous route

Lung infection of SARS-CoV-2 damages the epithelial barrier and endothelium, which facilitates the dissemination of the virus to circulation and various organ systems including the brain. Studies have shown strong evidence of RNAemia with plasma viremia in COVID-19 patients [22]. These studies have also demonstrated that SARS-CoV-2 viremia is a strong independent marker of COVID-19 disease severity and outcome. Viremia plays a role in broad tissue damage and persistent expression of several SARS-CoV-2 entry factors [22, 23]. An autopsy ultrastructural analysis of tissue from a COVID-19 patient revealed viral-like particles in endothelial cells, pericytes, and active budding across endothelium, strongly supporting hematogenous endothelial neuroinvasion [24].

#### Blood-brain barrier

ACE2 expression in microvascular endothelial cells of BBB provides an entry point for SARS-CoV-2 to the CNS. The virus may cross the BBB through paracellular, transcellular, or by a trojan horse mechanism. One animal study strongly suggested that the S1 protein can cross the murine BBB through adsorptive transcytosis [25]. Evidence from in vivo and in vitro BBB model studies demonstrated that SARS-CoV-2 crosses the BBB via transcellular paths utilising basement membrane disruption without evidence of tight junction alterations [26].

### Long-COVID and its neurological complications

#### Brain fog

‘Foggy brain state’ is the accepted term for a condition in which the cognitive functioning of an individual is not as sharp as usual. First formally described in the late 1980s and 1990s through reports of patients suffering from debilitating fatigue and significant cognitive dysfunction following the onset of an infection, a similar ‘brain fog’ has now been reported in many patients diagnosed with long-COVID [27, 28]. Initially this was termed chronic fatigue syndrome, however it lacked consensus among experts regarding its terminology, definitions, and importantly the criteria for diagnosis. Long-COVID is now undergoing a similar process in terms of definitions and diagnostic criteria, while also contending with the difficulties associated with including ‘brain fog’ as a symptom. Despite these difficulties a growing body of evidence suggests that in chronic fatigue syndrome there is central nervous system, autonomic nervous system, and immune system involvement, and there may be similar mechanisms at work in the long-COVID of today [29–31].

Although often lacking diagnostic clarity, brain fog is usually associated with lack of intellectual clarity, chronic mental fatigue, confusion, alongside short and long-term memory loss. Other symptoms include disability, dizziness, anxiety, reduced concentration, and impaired cognitive and mental abilities [32]. Brain fog has been reported in many conditions, such as hypothyroidism [33], menopause [34], coeliac disease [35], and now it has been closely associated with COVID-19 infection [36]. Diagnosis of brain fog often involves the patient reporting subjective experiences through symptom questionnaires and researchers or clinicians comparing that to objective performance-based tests used to evaluate patients with other cognitive abnormalities. Unlike the respiratory symptoms, which often become life-threatening, the neurological manifestations of COVID-19 are less noticeable to both patient and physician. Nonetheless, these manifestations are associated with significant disability, reductions in quality of life, and even mortality in COVID-19 patients [32]. Studies demonstrate that nearly 30% of COVID-19 patients experience symptoms such as the discussed ‘brain-fog’ [37] while also experiencing more concrete symptoms including delirium, stroke, and more noticeable cognitive impairments along with physical changes in the brain.

The neurological basis for the occurrence of brain-fog during long-COVID appears to be multifactorial. One potential factor involved is activities of the microglia, which are often implicated in neuroinflammation and neurodegenerative diseases [38–40]. This may occur as microglia express Toll-like receptors (TLRs) [41] which may get activated through damage-associated patterns (DAMPs). This process was recently implicated in the progressing of COVID-19 infection [42]. Another possibility is the hypothalmic-pituitary-adrenal axis (HPA axis) which controls the emotional state of an individual. This is typically activated by stress and has been seen to be activated by COVID-19 [43, 44]. It is interesting to note that microglia also express receptors for CRH and get activated when the stress level of the individual is elevated and this interaction has been implicated in the symptoms of COVID-19 [45]. Microglia may interact with mast cells in the brain resulting in their activation, which eventually leads to neuroinflammation [46–48]. Activation of mast cells and microglia in the hypothalamus leading to cognitive dysfunction is often seen in patients with mast cell activation syndrome, providing an example of how such an interaction may lead to the symptoms described here [49–51]. Psychological stress through CRH receptors directly activates the microglia [52, 53] and has pro-inflammatory effects leading to increased vascular permeability and disruption of the blood-brain barrier (BBB) [54, 55] possibly through the release of IL-6 [56], and CRH [57] which then further worsens the brain inflammation implied by the prior mechanisms by permitting the entry of even more viral particles, cytokines, and other toxic substances. It is now clear that damage to blood vessels and inflammation plays a role in the neurological symptoms described earlier. Additionally, no evidence of direct infection of the brain has been seen in COVID-19 [58], suggesting that the symptoms involved have a predominantly cytokine-mediated pathway. The microglia-activated neuroinflammation could explain both increased neurologic [59] and frequent psychiatric [60] disorders in patients with COVID-19, an increase which is also seen in patients with long-COVID syndrome [61].

#### Sleep disturbances

Altered sleep patterns are observed to be one of the most commonly reported neurological symptoms of long-COVID [62]. Huang et al. [63] reported that among the 1733 patients with long-COVID, 26% experienced sleep disturbances. In another study, investigators evaluated 251 COVID-19 survivors 1 month after hospital discharge, and among them 41.8% reported to have experienced insomnia at some point. Although this insomnia was improved among 25.5% of the initial population when re-evaluated 3 months post-discharge. The remaining patients however still experienced sleep disturbances marking a substantial portion of the initial study population [64]. Disturbances in sleep due to COVID-19 are of great concern because of bidirectional associations between mental health problems and sleep disturbance. This is likely a contributing factor for the mental health complications earlier described as related to COVID-19 [65]. Scarpelli et al. [66], argue that patients who have reported sleep disturbances, nightmares, and lucid dreaming could be suffering from long-COVID and that sleep disturbance are either directly a symptom or may be a reflection of the stress of the life-altering pandemic. In another study, 402 COVID-19 patients were assessed for insomnia 1 month after the hospital treatment and among them, 40% reported sleep disturbances [67]. Another follow-up study included 94 COVID-19 survivors who experienced COVID-19-related pneumonia with respiratory failure and when they tested for insomnia in 4th month of their discharge, 31% of them reported that they had insomnia [68]. In a similar follow-up study, 478 COVID-19 patients were evaluated for insomnia 4 months post-discharge and 53.6% of them reported they had insomnia [69].

Another observational follow-up study conducted on 797 COVID-19 survivors revealed that at 6 months of post-discharge, 4.9% of these patients have reported sleep disturbance [70]. However, another 6-month follow-up study that evaluated 796 patients found that at the time of assessment, 23.2% of these patients had neuropsychiatric symptoms such as a sleep disorder [71]. A study, conducted on 165 patients 6 months after their hospitalisation due to COVID-19 revealed that 31.5% of the patients had sleep disorders as one of the common neuropsychiatric symptoms [72]. Poor sleep quality was seen in 34.5% of 1142 COVID-19 patients who were evaluated for sleep quality at 7 months after their hospital discharge [73]. Another retrospective study reported that among the 23,6379 COVID-19 patients included in the study, 5.42% reported insomnia 6 months after the initial COVID-19 infection [74]. A prospective longitudinal study that assessed 1077 COVID-19 patients for sleep quality at 5.9 months after their hospital discharge found that 41.8% had worsened sleep quality along with other neurological symptoms [75]. A systematic review and meta-analysis that included 29 studies involving 13,935 patients revealed that the prevalence of sleep disturbance was 46%, poor sleep quality was 56%, the prevalence of insomnia was 38% and the prevalence of excessive daytime sleepiness was 14% [76]. Another systematic review (of 33 reports involving 282,711 participants with long-COVID) aimed to summarise the main psychiatric manifestations of long-COVID revealed that sleep disturbances, depression, post-traumatic symptoms (PTS), anxiety, and cognitive impairment were the common psychiatric manifestations in long-COVID patients after 4 weeks from COVID-19 infection recovery [77].

#### Memory impairment

A case series that addresses cognition in post-COVID-19 patients demonstrated evidence for neurocognitive deficits involving encoding and verbal fluency after severe COVID-19 infection [78]. Another case study involving a 62-year-old female without any psychiatric history developed psychiatric symptoms during COVID-19 infection and treatment, emphasising the correlation between the COVID-19 infection and confused state, short and long-term memory deficits, and delirium in patients as comorbidity, especially in elderly [79]. A case report from 3 patients identified neuropsychological symptoms such as inattention, executive function, and memory difficulties as potential post-infective complications [80]. Godoy-González et al. [81] recruited 80 critically ill COVID-19 survivors and conducted their cognitive and emotional assessment one year after hospital discharge which revealed that one-third of these patients were suffering from objective cognitive impairment with a frontal subcortical dysfunction. It was observed that female sex and post-traumatic stress disorder (PTSD) symptoms were predictive factors for the worst cognitive performance among these patients. A trajectory curve of memory loss in 2000 randomly selected COVID-19 patients revealed that memory problems are slower to recover compared to the brain fog and concentration loss, and suggests that memory loss may even be present for more than three years after the initial COVID-19 infection has resolved [82]. Such long-term data is of course limited by the comparatively short period during which COVID-19 has existed. Another systematic review and meta-analysis on long-term physical and mental sequelae of COVID-19 revealed that half of the survivors after the recovery have high life impact through either physical or mental sequelae for at least 12 months [83].

Imoto et al. [84] reported that long-COVID sequelae did not include production of sputum, dyspnoea, chest pain, sore throat, and diarrhoea but manifested with fatigue, dysgeusia, anosmia, alopecia, and sleeplessness. A recent case report suggests that COVID-19 infection can cause severe and selective neuropsychological impairments like prosopagnosia comparable to deficits seen after brain damage and it appears that long-COVID could also cause high-level visual impairments [85]. A study investigating the cause of cognitive decline in patients with long-COVID found that alterations in gut microbiome in these patients are also partly responsible for the long-term complications [86]. Recent case reports suggest a possible overlap between the long-term post-COVID symptoms and functional neurological disorders and they may share underlying biological mechanisms, such as stress and inflammation [87]. Tavares-Júnior et al. [88] studied the correlation between COVID-19, cognitive impairment, and APOE polymorphism in outpatients by conducting a cross-sectional study in COVID-19 patients in whom neurological symptoms that persisted more than three months from onset revealed that the group with cognitive decline exhibited a higher prevalence of the APOE ε4 allele than the normal group.

#### Neurodegeneration

The exact pathogenesis of neurodegeneration due to COVID-19 is yet to be elicited, and hypotheses include autoimmunity, persistent viral infection, chronic inflammation, and microbial dysbiosis [89]. Though neuroinvasive properties of SARS-CoV-2 via ACE2 receptors have been demonstrated, studies have failed to detect persistent viral RNA by RT-PCR technique directly from cerebrospinal fluid from those patients who manifested neuropsychiatric symptoms. When rarely detected, pathology lacks classic viral encephalitis pattern comprising clusters of inflammatory cells surrounding infected cells [90]. Instead, histopathology and magnetic resonance imaging of brains of patients who succumbed to COVID-19 showed punctate hyperintensities representing microvascular injury and fibrinogen leakage corresponding to basal laminar thinning of endothelial cells, and punctate hypointensities with relatively intact vasculature representing microhaemorrhages. There were perivascular-activated microglia, CD3+ and CD8+ T cells, macrophage infiltrates, and hypertrophic astrocytes in the perivascular spaces and in lumens adjacent to endothelial cells. These findings suggest multifocal microvascular injury, and are postulated to be due to glial activation and immune dysregulation rather than viral invasion [58]. These findings have further been demonstrated by single-cell transcriptome analysis from distinct areas of brain tissue using droplet-based single-nucleus RNA sequencing which confirmed microglial activation and CD8+ T lymphocyte infiltration, while failing to detect COVID-19 viral RNA [91].

In the acute phase, risk of vascular events are increased due to coagulopathy and endothelial inflammation [92]. The immunological differences have been noted to continue well beyond the acute phase, lasting more than a year, in individuals with long-COVID [89]. By using imaging mass cytometry on postmortem brain, Schwabenland et al. [93] identified pathognomonic microglial nodules with T cell infiltration in perivascular regions, with altered microglia-T-cell interactions and persistent immune activation. These translate to changes in population of inflammatory cell subset with increase in IL-4 and IL-6 secreting CD4+ T cells, while central memory CD4+ T cells and conventional dendritic cells are decreased [89]. This raises plausibility of ongoing neuronal damage through vascular dysfunction and compromised blood-brain barrier permeability, even in the absence of clinically apparent stroke [90].

Neuronal cell death has also been demonstrated following COVID-19 infection. In a pathological study [94], neuronal loss and changes in spatial arrangement in cerebral cortex was demonstrated using Voronoi tessellation. In larger series of 10 autoptic COVID-19 brains [95], cerebral cortex showed destroyed neurons within microglial nodule representing neuronophagia, along with axonal spheroids and disturbed radial architecture suggestive of axonal damage. This has also been demonstrated using human brain organoid model with forebrain-specific neural progenitor cells, showing SARS-CoV-2 not only infect neuronal cells, but can also promote death of nearby cells [12]. Studies on hippocampal neurons at subgranular zone and granule cell layer from patients who succumbed to COVID-19 showed fewer neuroblasts as compared to controls, suggesting that immature cell population was most affected, with pathophysiology linked to antineurogenic effects of IL-1β and IL-6 in hippocampus [96]. Apart from increased apoptosis and change in spatial distribution of hippocampal neurons, changes in morphological complexity of pyramidal cells with reduction in dendritic length and number of dendritic spine have also been noted [97].

Mouse model of mild respiratory COVID-19 infection established white matter-selective microglial reactivity and persistently elevated pro-inflammatory chemokines, such as CCL11, that persists up to 7 weeks post-infection. They have been associated with impairment of hippocampal neurogenesis and even myelin loss in subcortical white matter [98]. Identification of these cytokines provide biomarkers for prediction of long-COVID sequelae and opens up therapeutic options.

Apart from immune dysregulation with glial and vascular dysfunction, studies report that, in patients with COVID-19 infection, there is downregulation of ACE2 [99], which has been associated with tau hyperphosphorylation and augmented amyloid beta pathology resulting in Alzheimer disease pathology in animal models [100]. A study conducted by Charnley et al. [101] identified two strongly amyloidogenic sequences from SARS-COV-2 proteome, which when screened against neuronal cells revealed their toxic nature similar to Alzheimer disease pathology. The protease-resistant structure and cytotoxicity of these assemblies is worrying, as its potential to trigger progressive neurodegeneration remains uncertain, and will only be evident over time in epidemiological studies.

The impact of neurodegeneration through various mechanisms mentioned above can be measured through biomarkers, clinical evaluation, and radiological markers. In a meta-analysis involving 2,049 people, dominant cognitive decline was noted in domains of executive function, which is anatomically localised to the prefrontal cortex involving the executive network [102]. Work done by Baseler et al. [103] using a working memory quiz on patients with COVID-19 sequelae detected impairment in recalling a sequence of digits just heard, suggesting dysfunction of memory encoding and retrieval processes, anatomically localising to hippocampus and medial temporal lobe. Radiological correlation of the same can be observed in a longitudinal imaging study of brain structure in UK Biobank [104], wherein participants had an initial scan prior to infection. Detrimental effect of COVID-19 infection was mainly noted in olfactory cortex and limbic system, along with distinct reduction of grey matter thickness in lateral orbitofrontal cortex and left parahippocampal gyrus. These findings were also noted by Fraser et al. [105], wherein there was an additional loss of 0.7% in olfactory related brain region in post COVID-19 infected participants, which was larger on comparison with population-based studies of hippocampal volume which detected longitudinal loss of only 0.2–0.3% per year.

Pooled analyses from recent larger meta-analysis [106] including twelve studies and 33 million individuals, of which more than 2.5 million were post-COVID-19 cases, showed increased risk for dementia (HR = 1.66, 95% CI 1.42–1.94, *I*^2^ = 91%), new-onset Alzheimer’s disease (HR = 1.50, 95% CI 1.22–1.85, *I*^2^ = 97%), and Parkinson’s disease (HR = 1.44, 95% CI 1.06–1.95, *I*^2^ = 86%) among COVID-19 survivors, further strengthening the association of higher risk for neurodegeneration.

### Implication of long-COVID in chronic neurological disorders

#### Alzheimer’s disease

The key question is whether the neurological symptoms of post-COVID is self-limiting or progressive, and does the infection accelerate the course of the existing dementia. Identifying this association is difficult, as multiple factors could contribute to the cognitive decline including those directly resulting from COVID-19 infection such as severity, preexisting medical comorbidities (including, vascular events, obesity, hypertension, diabetes), and treatment parameters comprising remdesivir, steroids, benzodiazepines, monoclonal antibodies, and other experimental therapeutic strategies [107]. In the acute setting of COVID-19 infection, approximately 28% of patients aged 65 and above admitted to emergency care was in delirium [108], which itself is a forerunner of long-term cognitive consequences, such as increased risk for developing Alzheimer’s disease [109, 110]. Another postulate was the effect of confinement during the pandemic which could lead to rapid deterioration in cognitive reserve of patients with dementia. However, studies conducted in Spain [111] and China [112] showed no progression in Alzheimer’s or other dementia due to this “lockdown effect”.

Susceptibility to COVID-19 transmission in Alzheimer’s dementia patients have been postulated due to increased ACE2 expression noted in them in genome wide association studies [113]. Moreover, following SARS-CoV-2 viral invasion, there is a notable down-regulation of these ACE2 receptors [114], which has been correlated with significant increase in levels of phosphorylated tau and Aβ pathology [100]. SARS-CoV-2 infection significantly upregulates the expression of interferon-induced transmembrane protein 3 [115], which enhances the γ-secretase activity, which cascades to rise Aβ deposition [116]. Worrisomely, Vavougios et al. [117] was able to demonstrate using above complexes in comparative transcriptomic studies, that a feed-forward signal is initiated which could propagate amyloid pathology, even after clearing infection. Recent research [118], utilizing mouse model overexpressing human wild type amyloid precursor protein (APP) and human brain organoids, reported that the APP interacts with the spike protein of SARS-CoV-2, enhancing cellular entry of the virus, and exacerbating Aβ-associated pathology. They also noted that these changes can be ameliorated by N-terminal APP blockage, thus paving the way for therapeutic strategies.

Noteworthily, COVID-19 and Alzheimer’s disease share several risk factors including APO ε4 expression [119], besides age, gender, hypertension, and diabetes. As previously noted, the role of persistent neuroinflammation and cytokine storm which reduces microglial phagocytosis of Aβ amyloid [120], blood brain barrier dysfunction with loss of pericytes which impair clearance of Aβ peptides [121], and cerebral hypoperfusion accelerating phosphorylation rate of tau [122], may all contribute synergistically to progressive Alzheimer’s pathology. Together, they all result in neurodegeneration, which has also been substantiated by increase in levels of various biomarkers, such as neurofilament light chain and hyperphosphorylated tau, in cerebrospinal fluid of patients following COVID-19 infection [123–125].

Indirect effect of behavioural and psychiatric manifestations of long-COVID on the cognitive decline also requires mention. Animal studies have showed depression-like stress worsens progression of Alzheimer’s disease [126], and PTSD-like induction accelerates the accumulation of Aβ through disruption of the corticotropin-releasing factor [127]. As previously highlighted, sleep disorders in post COVID-19 patients, also negatively impact cognitive function [128, 129]. Tackling these disorders through therapy and pharmacological interventions, might help improve the quality of life, whilst reducing the additive effects on dementia progression.

The central role of inflammation in various pathophysiological processes leading to cognitive decline is evident and this forms the basis for immune regulation and improving microcirculation as therapeutic strategies [130]. While awaiting these developments, clinical assessment and multimodal neuroimaging mediated targeted cognitive rehabilitation must be offered.

#### Parkinson’s disease

The relation between COVID-19 and Parkinsonism is complex, and many uncertainties remain. While there is insufficient evidence to show that Parkinsonism by itself increases the risk of acquiring COVID-19 infection [131], its converse, the idea of COVID-19 unveiling Parkinsonism is worrying, fear of which is mainly fuelled by consequences of encephalitis lethargica in early 20th century, which still remains as a medical mystery, with many researchers associating its etiology to Spanish influenza virus [132].

Acute worsening of motor and non-motor symptoms has been noted following contracting SARS-CoV-2 infection. Mechanisms suggested include infection-induced altered dopaminergic neurotransmission [133, 134], pharmacodynamic and pharmacokinetic changes to dopaminergic medication, and infection related secondary neurodegeneration [135].

In a multicentre case series consisting 27 patients with Parkinsonism affected by COVID-19 [136], 85% developed prolonged symptoms with the most common effects being worsening of motor symptoms (52%), augmented levodopa dosage needs (48%), fatigue (41%), and cognitive disturbances (22%). Postulated theories behind these findings were persistent systemic inflammation [137] and viral illness associated worsening of preexisting Parkinsonism, besides strain of prolonged lockdown, and limited access to rehabilitation services [136]. In another series by Brown et al. [138], among patients with Parkinsonism and COVID-19, 63% complained of motor symptoms, and 75% of nonmotor symptoms, whilst among patients with Parkinsonism without COVID-19, 43% noted worsening in motor symptoms and 52% worsening in non-motor symptoms, which were attributed to disrupted medical care (64%), social activities (57%), and exercise (21%).

Around 20 cases of new-onset Parkinsonism related to diagnosis of COVID-19 have been reported [136]. In majority of them, Parkinsonism occurred in context of encephalopathy, while four patients presented with symptomatology suggestive of typical idiopathic Parkinson’s disease (PD). Functional dopaminergic scans in few cases revealed decreased uptake in either or both putamina. Though virus could not be detected in CSF and screening for known antibodies were negative, an immune-mediated pathology was suspected with neuroinflammation and subsequent cytokine storm targeting midbrain dopamine neurons, made vulnerable due to its increased metabolic demands [139, 140]. SARS-CoV-2 pandemic with its restrictions, and long-COVID sequelae have had a clear impact on patients with Parkinsonism. Multifactorial worsening in symptomatology may have had negative consequences, including gait dysfunction and increased risk of falls, having short- and long-term impact on quality of life and healthcare burden.

### Long-COVID and psychological, psychosocial aspects and quality of life

The COVID-19 pandemic has had significant direct and indirect impact on the psychosocial aspects of the society. During the initial phase of COVID-19 spread, coronaphobia generated a plethora of psychological manifestations across different strata of society across the globe. After even mild episodes of COVID-19, patients infected with coronavirus reported experiencing significant persistent symptoms that considerably reduced their quality of life for months which is often referred to as ‘long-COVID’. Psychological changes associated with long-COVID are well established. Psychological distress, assessed at the beginning of the COVID-19 pandemic, was linked with having high risk for persistent psychological symptoms after being infected with coronavirus [141]. A longitudinal study has established a link between beliefs about COVID-19; such as one’s estimated symptom severity (if infected) and their perception of the body’s ability to fight diseases with the symptoms experienced weeks afterward [142]. Symptom expectations associated with coronavirus infection and self-reported history of COVID-19 were reported to be more predictive of worsening of somatic symptoms than serology test results during the pandemic. However, such studies did not provide a comprehensive picture of the psychological mechanisms involved. Rather, they postulate that there may be some persistent symptoms in a few patients which are likely to be influenced by psychological factors [143].

Apart from the common neurological symptoms and complications, people with long-COVID reported impaired quality of life, mental health and employment issues [144]. Furthermore, patients suffering with long-COVID tend to experience exercise intolerance, impaired daily functioning and poor quality of life [145]. A study conducted among patients with persistent COVID-19 symptoms for more than 6 months after a mild COVID-19 infection found that prevalent symptoms were significantly linked with poor long-term health, quality of life, and psychological distress [146]. Shachar-Lavie et al. [147] found that long-COVID might affect academic performance of children due to it’s association with impairments in aspects of children’s memory.

Recent studies have estimated the extent to which long-COVID affects the cognitive functioning of an individual and its outcomes. Long-COVID often presents with “brain fog”, characterised by decreased energy, concentration issues, and disorientation [148]. Similarly, another study reported that loss of smell, poor concentration, and insomnia were persistently reported by individuals during long-COVID and that this resulted in reduced quality of life [149]. Another study investigated the effects of mental clouding on olfactory functioning and found that mental clouding tends to affect an individual’s ability to identify odours in turn affecting the olfactory function. This established a positive correlation between mental clouding and olfactory function. Moreover, people with long-COVID who were reported to have either mental clouding, headache, or both were found to have severe olfactory dysfunction compared to those without any neurological complaints [150].

#### Anxiety, depression, and altered mental status

The psychological manifestations of long-COVID of which anxiety and depression are the most studied. A positive correlation has been established between levels of depressive symptoms and anxiety in a long-COVID non-hospitalised cohort [151]. Cognitive complaints were also found to be associated with both anxiety and depressive symptoms exhibited by patients with long-COVID [152, 153]. A study conducted by Calabria et al. [154] reported that higher levels of apathy, executive dysfunction in neuropsychiatric measures, and anxiety were found to be important predictors among different types of fatigue such as physical, mental and psycho-social exhaustion and were likely to have an impact on the daily functioning of persons.

Many COVID-19 patients and survivors suffer from various neuropsychiatric disorders. Symptoms such as attention deficits, anosmia, depression, anxiety, psychosis, seizures, and suicidal tendencies have been observed during and after SARS-CoV-2 infection [155]. Neuropsychiatric issues associated with COVID-19 may be linked to the neurotropism of the virus. A previous study examined 18 brain samples from patients who died within 32 days after infection and showed no specific brain changes or encephalitis apart from hypoxic changes. The immunohistochemical staining did not reveal cytoplasmic viral particles in either neurons or glial cells [156]. In contrast, evidence from another study suggests that neuroinvasion of the virus occurs via the neuro-mucosal interface into the olfactory tract of CNS. Therefore, the role of non-neuronal pathways in the entry of SARS-CoV-2 into the brain cannot be neglected [14].

**Fig. 3:**
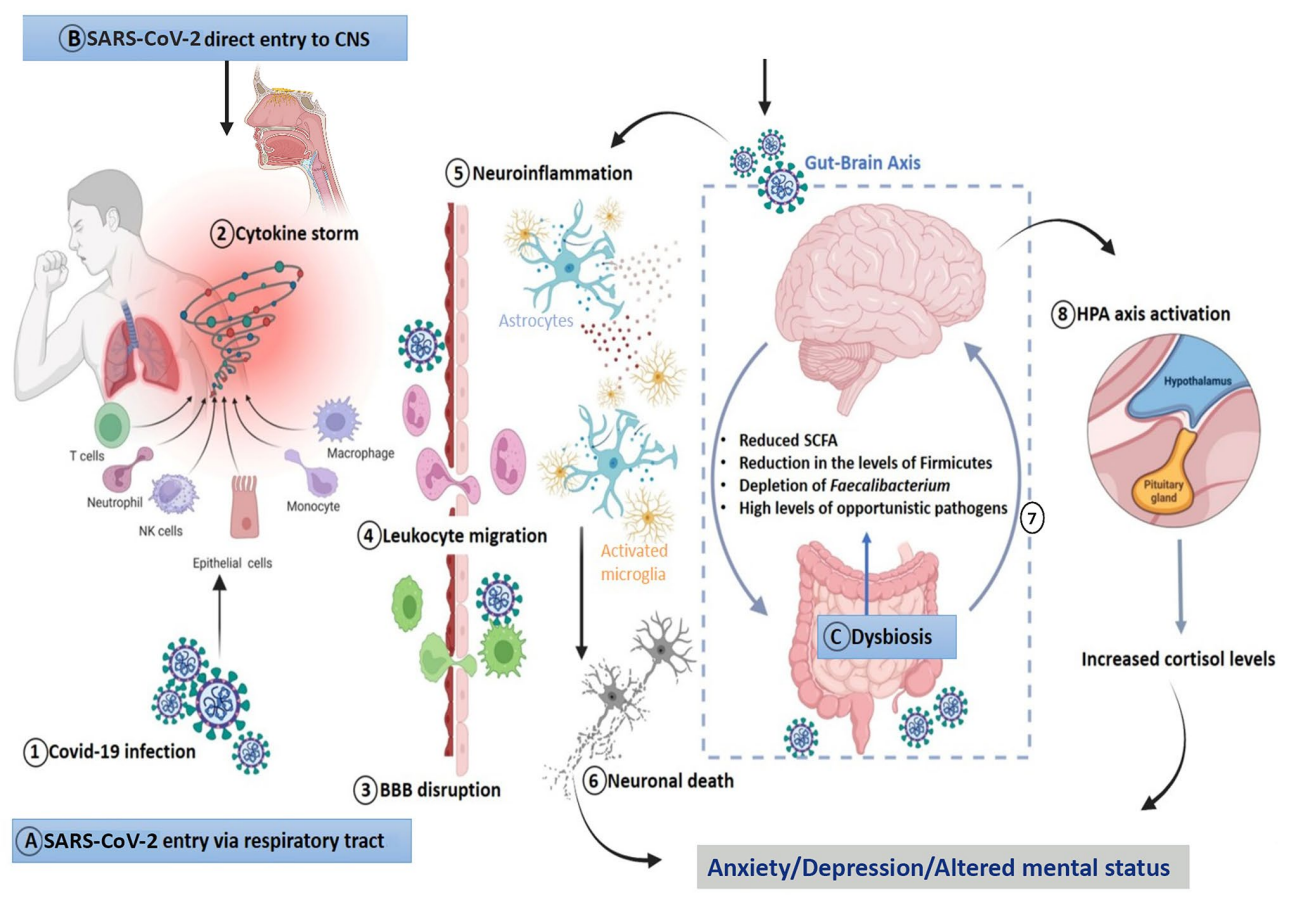
Plausible mechanisms for anxiety, depression and altered mental status. (**A**) Infection via the respiratory tract: (1) COVID-19 infection associated upregulated levels of proinflammatory cytokines, (2) Cytokine storm damages organs, including the brain, (3) Disruption of BBB, (4) Damaged BBB enables migration of leukocytes into the brain, (5) Activation of microglia and astrocytes, increases neuroinflammation, (6) Neuronal damage and altered connections lead to anxiety and depression; (**B**) Direct viral entry into the brain via the olfactory tract cause the similar cascade of events described earlier; (**C**) Gut dysbiosis: COVID-19 infection induced gut dysbiosis can cause neuroinflammation, leading to anxiety and depression, (7) Altered gut-brain axis and neuroinflammation lead to (8) HPA axis activation and resulting increased cortisol levels has a role in anxiety and depression. *Image created using BioRender*

Long-COVID related neuropsychiatric attributes may persist even after months of acute infection including anxiety, depression, suicidal ideation, and mood shifts [157]. It is reported that nearly 1.3 million individuals showed COVID-19 related anxiety and depression, which then returned to normal over time, and that elevated risks of cognitive impairment or neurocognitive conditions persisted for at least 2 years [109, 158]. The possible mechanisms underlying the neuropathologies discussed include neuroinflammation, coagulopathy induced vascular damage, endothelial dysfunction, and neuronal damage [109] (Fig. 3). The direct effect of viral entry into the brain or the increased inflammatory responses of the body to the virus may have altered the neuronal signalling or connections that resulted in such cognitive changes [159] (Fig. 3).

The exact cause of anxiety, depression and/or altered mental status is still unknown. However, mood disorders have been linked to alterations in neurotransmitters such as serotonin, dopamine and norepinephrine. There is also growing evidence that the dysregulation in the hypothalamus-pituitary-adrenal (HPA) axis leads to the production of a higher level of cortisol, which could be associated with an elevated risk of depression and anxiety [160]. There is an undeniable role of inflammation in mood disorders through the action of increased levels of inflammatory cytokines [161]. When there is infection or injury, blood brain barrier disruption may occur, which can cause the entry of peripheral immune cells to the CNS. This leads to an increased level of pro-inflammatory cytokines, interleukin 1β, tumor necrosis factor ∝, interleukin 6, and chemokines which in turn cause activation of microglia and astrocytes. Microglial activation and astrogliosis leads to neuroinflammation [162] (Fig. 3). These cytokines can activate the kynurenine pathway, which interrupts serotonin production. The subsequent activation of the HPA axis can then lead to increased production of the stress hormone cortisol [163]. This neuroinflammation can cause detrimental effects to specific brain regions such as, the amygdala, hippocampus, hypothalamus, and cerebral cortex, all of which have a role in regulating emotional responses (Fig. 3). The alteration in cytokine signals also regulates neurotransmitter metabolism, neuroendocrine function, and mood-regulating neurocircuitry, contributing to neuropsychiatric disorders [164]. Studies suggest that ‘cytokine storm’ or elevated levels of pro-inflammatory cytokines such as TNF-α, IFN-γ, IL-6, IL-8, IL-10, IL-16, IL-17A, and IL-18 associated with severe COVID-19 infection persist even after the recovery from the infection [165]. Additionally, it has been reported that the neuroinflammation caused by the cytokine storm that occurs during the active infection phase of COVID-19 results in the new onset of mood disorders [166]. The increased cytokine response associated with COVID infection may communicate with the CNS when there is a disruption in the BBB. It can lead to the activation of microglia and interleukins, which causes increased cytokine levels. These events may lead to oxidative stress, neuroinflammation, excitotoxicity and neural tissue damage [167].

The gut and brain are connected by a bidirectional link known as ‘gut-brain axis’ which is thought to play an inevitable role in cognition via various pathways [168]. There is a close relationship between the alteration in gut microbiota or ‘dysbiosis’ and several mood disorders, such as anxiety and depression [169, 170]. Most of the gut microbiome (more than 70%) consists of the phyla Firmicutes and Bacteroides. A small number of Proteobacteria, Actinobacteria, Fusobacteria, and Verrucomicrobia are also present [171]. Microbial metabolites from these bacteria are considered the central modulators of the gut-brain axis and have been shown to play a central role in the development of depression [172]. A study on patients with generalised anxiety disorder found a marked reduction in microbial richness with a distinct metagenomic composition. Fewer short-chain fatty acid (SCFA) producing bacteria were present in the patient’s gut than in healthy controls [169]. Similarly, a previous study on patients with major depressive disorder showed fewer Bifidobacterium and Lactobacillus [173]. These beneficial gut microbes produce metabolites like SCFAs, specifically acetate, butyrate, and propionate, which can modulate neurotransmitters, neurotrophic factors, and neuroinflammation [174] (Fig. 3).

A similar change in the gut microbiome associated with COVID-19 infection has been reported widely. Dysbiosis was detected in COVID-19 patients with significant changes in the abundance of beneficial bacteria [175]. A study on hospitalised patients reported a significant reduction in Firmicutes and depletion of Butyrate-producing bacteria; Faecalibacterium [176]. The SCFAs have a role in maintaining gut integrity by acting as an energy source for colonocytes and regulating inflammatory responses via various signalling mechanisms like NF-кB signalling [177]. The reduction in microbial richness with a drop in SCFA-producing bacteria and an increase in opportunistic bacteria could be associated with the COVID-19 associated neuropsychiatric disorders [174]. The “long-COVID” condition of the GI tract may be an underlying cause of mood changes, anxiety and depression in the post-COVID scenario (Fig. 3). The link between gut dysbiosis and the neuropsychiatric disorders in long-COVID is uncharted and warrants further investigation.

### Plausible pathophysiology of neurological and psychological complications

**Fig. 4:**
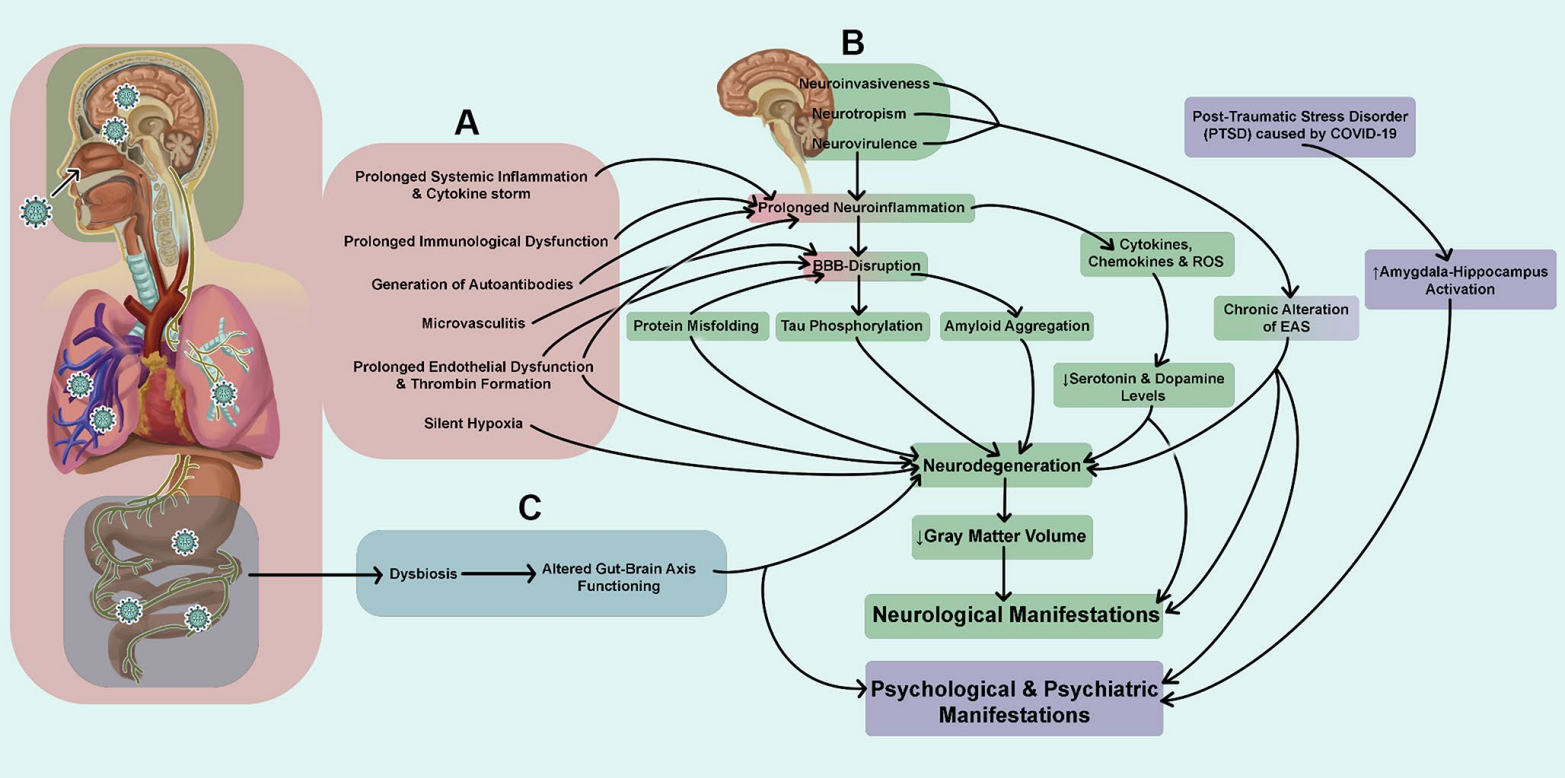
Plausible comprehensive pathophysiology for neurological and psychological complications associated with long-COVID syndrome. Persistent systemic and various organ system-associated changes could be the possible basis for the development and progression of long-COVID associated symptoms. These may include but are not limited to persistent changes that occur in the whole body (**A**), changes that occur specifically in the CNS (**B**), and changes that occur in the GUT (**C**). As depicted in this figure, all of these changes and effects associated with them show dramatic interrelations and interdependence in long-COVID patients

As multiple factors are involved in long-COVID syndrome, the overall pathophysiology of the neuropsychiatric and psychological complications during long-COVID syndrome are also multifaceted. Persistent systemic and various organ system-associated changes could be the possible basis for the development and progression of these complications in long-COVID patients (Fig. 4).

*a) Neuroinvasiveness*, *Neurotropism and Neurovirulence*: Neuroinvasiveness is the ability of coronavirus to enter the PNS and CNS. Neuroinvasiveness gives this virus an edge for inducing infection and facilitates replication in cells of the nervous system including neurons, glial cells, microglial cells, meningeal cells, choroid plexus cells, cellular components of neurovascular system such as vascular endothelial cells and pericytes (Neurotropism). Neurovirulence is the ability of coronavirus infection to cause pathology in the nervous system contributing to the development of clinical disease of the CNS independently of its ability to invade or infect cells of the CNS [178].

*b) Persistent systemic inflammation and cytokine storm*: It is now evident that coronavirus infection induces irregularities in both innate and adaptive immunity. These abnormalities may include monocyte expansion, T-cell exhaustion, and significantly prolonged cytokine release leading to tenacious neuroinflammatory responses including activation of microglia. This is postulated to be one of the causes of white matter abnormalities and microvascular changes in the brain of long-COVID patients [179, 180].

*c) Prolonged neuroinflammation*: This is the chronic inflammatory response seen in the brain and spinal cord. Neuroinflammation is reported to be mediated by the persistent production of cytokines, chemokines, reactive oxygen species (ROS), and second messenger systems in patients with long-COVID [179, 181]. Normally there are immune, physiological, biochemical, and psychological consequences to neuroinflammation [182] and this could be even worse in persistent neuroinflammation [179] leading to the neurological issues of long-COVID.

*d) Blood-brain barrier disruption*: It is reported that coronavirus could disturb the BBB integrity and lead to derangement of tight junctions or adherens junction proteins. This could lead to increased permeability of the BBB, leading to leakage of blood components and movement of immune cells into the brain parenchyma [179, 183].

*e) Generation of autoantibodies*: Reports have suggested the presence of autoantibodies targeting the brain in the blood and CSF of critically ill COVID-19 patients. This, could lead to neurological symptoms in long-COVID [184].

*f) Microvasculitis*: Reports indicates that persistent systemic inflammation during long-COVID leads to microvasculitis in the brain [185, 186] which could potentially be another possible mechanism involved in the neurological consequences.

*g) Prolonged endothelial dysfunction*, *platelet activation*, *and enhanced thrombin generation*: Persisted elevated levels of D-dimer, FVIII, thrombin, vWF, ICAM-1 and IL-6 were found in patients 1 year after the recovery from COVID-19 infection [187]. Endothelial dysfunction [188], hypercoagulable conditions, and the associated microthrombosis [189, 190] in the brain vasculature could be other reasons for the altered functioning of brain regions in long-COVID [191].

*h) Silent hypoxia and associated issues*: Silent hypoxia has been reported in several COVID-19 patients [192]. This is a condition where an individual has 50–80% oxygen saturation levels where a 95% or higher is expected, but the individual does not experience any breathing difficulties. Silent hypoxia is deleterious for the brain as this may directly contribute to brain damage in addition to encouraging ctyokine storm by engaging various mediators of inflammation and inducing significant endothelial injury through NF-κB transcription factor activation. Hypoxia and metabolic disturbances can significantly damage neurons and other brain cells [193].

*i) Dysregulated levels of neurotransmitters like serotonin and dopamine*: Recent evidence suggests that decreased levels of serotonin and dopamine could contribute to the development of long-COVID. In one study, although viral inflammation did not change the brain serotonin level, the reduction in peripheral serotonin levels impeded the activity of the vagus nerve resulting in changes in hippocampal responses and memory [194]. Reports also suggest the possible modulation of dopamine (DA) homeostasis during long-COVID resulting in neurodegenerative diseases. Proinflammatory molecules (cytokine, chemokine, and ROS) could modulate DA homeostasis leading to neurological abnormalities [195].

*j) Amyloid aggregation*: Several viral proteins are reported to be amyloidogenic. Amyloid precursor protein expression (APP) is found to be upregulated after coronavirus binding to ACE2 receptors [196]. Camacho et al. [197] identified six upstream regulators which increased APP production in COVID-19 patients and RNA-seq analysis revealed increased APP transcripts in blood samples of COVID-19 patients compared to people without COVID-19 [198]. Recently, Nyström et al. [199] have proposed a molecular mechanism for potential amyloidogenesis of coronavirus S-protein in humans facilitated by endoproteolysis. This could be the basis for pathogenesis of neurodegenerative symptoms in long-COVID.

*k) Tau phosphorylation*: Reports suggest that translocated lipopolysaccharides or microbial DNA can induce Tau hyperphosphorylation which could contribute to memory problems. One of the central markers of tauopathy is pTau (Tau hyperphosphorylation). Apart from aggregated long fibrils, recent data suggest that short pTau is associated with peripheral neuropathy in COVID-19 [4]. Reiken et al. [200] observed evidence of oxidative stress and corresponding activation of inflammatory pathways leading to leakage of Ryanodine receptor (RyR). This resulted in unregulated intracellular calcium levels, leading to activation of calcium-dependent enzymes and hyperphosphorylation of tau proteins in autopsy tissue samples of patients with COVID-19 compared to controls.

*l) Protein misfolding*: Protein misfolding is one of the most studied causes of neurodegenerative diseases. An incident of Creutzfeldt-Jakob disease (CJD) in a patient two months after mild COVID-19 has been reported and suggested to have been associated with a neuroinflammatory state encouraging protein misfolding and subsequent aggregation of misfolded form (PrPSc) of prion protein (PrPc) [201]. The role of this in long-COVID is a possibility and requires further research. Another report shows that coronavirus-related exosomal cargo contains modified (misfolded) host proteins and inflammatory mediators in addition to coronavirus RNA and proteins which could potentially promote neurodegenerative and neuroinflammatory cascades leading to Parkinsonism and PD development [202].

*m) Neuronal death*: Coronavirus-induced astrogliosis and microgliosis are the two mechanisms that could be attributed to the possible cause for neuronal damage in long-COVID [203]. Furthermore, GnRH-secreting neuronal death is reported in post-mortem brain samples of patients infected with COVID-19 which dramatically reduces GnRH expression in the hypothalamus. Evidence suggests that hypothalamic GnRH system neuroinvasion by the virus could underlie certain post-COVID symptoms such as augmented cognitive decline [204].

*n) Changes in grey matter volume*: Recent evidence suggests that COVID-19 infection could alter cortical grey matter volume in humans [205]. Compared to normal age-matched controls, decreased cortical grey matter volume was observed in 24 COVID-19 patients [205]. However, further research is required to determine the exact mechanism that cause a reduction in cortical grey matter volume in humans.

*o) Prolonged immunological dysfunction*: Recent research reveals bilateral cortical alteration in prefrontal cortices, temporal gyri, insulae, posterior cingulate, and parahippocampal gyri in both COVID-19 survivors and patients with long-COVID and significant cognitive impairment. This correlated with significant increase in circulating cytokine levels in patients with long-COVID who have cognitive impairments compared to healthy never-infected controls [206]. Ceban et al. [207] have also reported that persistent immunological dysfunction post-COVID is prevalent in patients and is associated with long-COVID neurological or psychiatric symptoms. Recently, Yin et al. [208] also reported altered concentrations of TNFα, IL-1b, and IL-6 in long-COVID.

*p) Dysregulation of the cholinergic system*: Evidence suggests that SARS-CoV-2 virus can disrupt cholinergic system activity. The effects include, but are not limited to, myasthenia gravis, and altered acetylcholine activity [209]. The mechanism for this arises from sequential data of the coronavirus spike protein, as it is reported to have a similar sequence of neurotoxins, and is capable of binding to, nicotinic acetylcholine receptors (nAChR). The implications of this in long-COVID is yet to be explored. Since the vagus nerve is among the neuroinvasion pathways of coronavirus, dysregulation of the cholinergic system by coronavirus demands further investigation.

*q) Chronic alteration of the extended autonomic system including neuroendocrine and neuroimmune systems*: The extended autonomic system (EAS) consists of the central neural autonomic network, autonomic nervous system and several neuroendocrine systems [210]. Long-term EAS activation in COVID-19 patients may lead to regulatory system disruption [211] and this may be another possible mechanism in the development of long-COVID. On the other hand, hypocortisolism is observed in long-COVID patients. This is associated with depression and anxiety levels comparable to the clinical features observed in myalgic encephalomyelitis. It is believed that the failure of the hypothalamic-pituitary-adrenal axis to recover following an acute illness is due to protracted stress which could be responsible for the above-discussed observations and associated clinical manifestations in long-COVID [212].

*r) Altered gut-brain axis functioning*: As discussed earlier persistent inflammation is associated with changes in the gut microbiome and alterations in neuro-immune interactions. Research has revealed an association between GI symptoms and abnormal cognitive assessments and sleep disturbances of long-COVID patients [213]. Significant numbers of studies have demonstrated strong links between dysbiosis in the gut and neurodegenerative and neurodevelopmental diseases [214]. Reports about metagenomic sequencing studies of stool samples have demonstrated a direct correlation between fatigue and brain fog with an intestinal reduction of certain groups of bacteria and increased percentage of certain others in patients with long-COVID [215]. Moreover, respiratory and neuropsychiatric symptoms of long-COVID are directly proportional to the presence of intestinal nosocomial pathogens [86].

*s) Post-traumatic stress disorder*, *increased amygdala and hippocampal activity*: Many COVID-19 survivors have shown significant mental health issues such as post-traumatic stress disorder, anxiety, and depression. Neuroimaging studies have revealed increased amygdala activity in male survivors of COVID-19 and hippocampal activity is strongly correlated with depressive symptoms in this group [216]. However, further research is needed to understand the involvements of these mechanisms (activation of amygdala, and hippocampus, etc.) in long-COVID.

### Management of neurological complications in long-COVID and recommendations

For those with long-COVID symptoms, there are currently only limited evidence-based treatments addressing neurological complications. Most management options are based on experience on cognitive recovery following sepsis [217]. Patients may benefit from reducing existing cardiovascular risk factors, optimizing nutrition, adhering to structured exercise programs, practicing proper sleep hygiene, attending physical/occupational and cognitive therapy, joining support groups [218], and seeking mental health support [219]. Clinicians must be aware of delayed or lingering cognitive symptoms and perform neuropsychological evaluation where needed. Regularising follow-up, facilitation of referrals to specialists, and coordination across health services may help mitigate long-term outcomes.

Multiple treatment modalities have been tested to counter the cognitive deficits present in long-COVID patients. Pooladgar et al. [220] reported that 5 mg donepezil did not improve memory in patients with post-COVID memory impairments but could exert a positive effect on specific memory subsets. Another report demonstrated that administration of *Ginkgo biloba* special extract (2 × 80 mg daily) improved and completely restored cognitive deficits in long-COVID patients with cognitive deficits suggesting a potential low-risk treatment option in these patients [221]. It has been reported that a regimen including oral palmitoylethanolamide and luteolin along with olfactory training ameliorated the olfactory dysfunction and memory problems in patients suffering from long-COVID [222]. A recent case report also demonstrates that accelerated Theta-Burst Transcranial Magnetic Stimulation (TBS) over the bilateral dorsolateral prefrontal cortex (DLPFC) ameliorated long-COVID-related neuropsychiatric symptoms [223].

Pharmacologically, recent meta-analyses showed protective role of targeting renin angiotensin system [224], probably by ACE2 mediated inflammatory and ischaemic pathways. Following this, trials testing angiotensin receptor blockers such as losartan in COVID-19 patients have been registered. Approaches exploring other inflammatory targets such as TMPRSS2, TLR4 blockers and ADAM-17 inhibitors are also underway.

Meanwhile, long-COVID patients feel frustrated to see these medical research findings not being translated for their benefits. Repurposing already approved medications as a strategy for early intervention revealed that Furosemide, besides functioning as a loop diuretic, also has role in reducing proinflammatory cytokines such as TNF-*α* and IL-6, with ability to regulate different microglial phenotypes [141], thereby suggesting potential in treatment of Alzheimer’s pathology. There are previous studies demonstrating effect of loop diuretics for reducing risk of dementia [225, 226], which presents an interesting opportunity for further development of these molecules to treat neuroinflammation. UK-based STIMULATE-ICP trial is enrolling participants into study arms for colchicine, rivaroxaban, and famotidine, compared to placebo [227]. There are more than 300 clinical trials that enrol patients with long-COVID; around 200 of which are interventional studies. Most treatments target immune cell suppression or modulation. Some pharmacological and non-pharmacological clinical trials are represented in Table 1. The oncoming burden of long-COVID awaiting healthcare system and economy is huge, and this calls for better diagnostic methods and stratification, along with powerful clinical trials for therapeutic options. The effects of long-COVID, including persistent chemical sense disorder-associated psychiatric disorders [228] in children and teenagers are comparatively less studied and reported. Hence, future studies must also include these sensitive populations which will help us understand the mechanisms that underlie long-COVID associated neurological, neuropsychiatric, and psychological changes in them.

**Table 1:** Pharmacological and non-pharmacological interventional clinical trials on long-COVID

**Pharmacological interventional trials**	
A phase IIa double-blind, randomized placebo-controlled trial of tocilizumab to investigate the effect on health-related quality of life in adults with long-COVID and persistent inflammation	ISRCTN46454974; ISRCTN, UK
Feasibility study of amantadine for cognitive dysfunction in patients with long-COVID: A pilot randomized control trial	NCT06234462; ClinicalTrials.gov, USA
STIMULATE-ICP: A pragmatic, multi-centre, cluster randomised trial of an integrated care pathway with a nested, phase III, open-label, adaptive platform randomised drug trial in individuals with long-COVID - Loratadine + Famotidine / Colchicine / Rivaroxaban	ISRCTN10665760; ISRCTN, UK
A prospective interventional study to assess role of an integrated approach of homoeopathy along with WHO rehabilitation self-management care for long-COVID: Single blind placebo controlled randomized trial	CTRI/2023/03/050878; CTRI, India
A randomized control trial to evaluate the efficacy of ayurvedic interventions (Agastya Haritaki and Ashwagandha) and yoga in long term effects of COVID-19 - POST COVID	CTRI/2021/03/031686; CTRI, India
An open label clinical study to evaluate the safety and efficacy of molecular medicine, CelWel - Natural extracts of *Tinospora cordifolia* (common herb with the local name guduchi) combined with pepper extracts	CTRI/2022/12/048144; CTRI, India
**Non-pharmacological interventional trials**	
Testing non-pharmacological Traditional Chinese Medicine (Acupuncture) based treatments for cognitive impairment in people with long- COVID symptoms: A randomized controlled trial	NCT06042777; ClinicalTrials.gov, USA
Transcranial direct current stimulation as a strategy for the management of disorders generated by COVID-19: A multicentric study.	NCT06074848; ClinicalTrials.gov, Brazil
Amygdala insula retraining in the management of long-COVID symptoms	NCT05851846; ClinicalTrials.gov, USA
Remote diet intervention to reduce long-COVID symptoms trial	ISRCTN12595520; ISRCTN, Scotland and UK
A mobile intervention merging yoga and self-management skills (MY-Skills Mobile) for individuals experiencing symptoms of long-COVID-19	NCT05697042; ClinicalTrials.gov, USA
Interventions to teach mindfulness skills for persisting symptoms of COVID-19: Minimizing impact of symptoms on everyday functioning and on healthcare usage/utilization	NCT05268523; ClinicalTrials.gov, Canada
